# Impact of delirium on the outcome of stroke: a prospective, observational, cohort study

**DOI:** 10.1007/s00415-022-11309-2

**Published:** 2022-08-09

**Authors:** Eleonora Rollo, Valerio Brunetti, Irene Scala, Antonio Callea, Jessica Marotta, Catello Vollono, Giovanni Frisullo, Aldobrando Broccolini, Paolo Calabresi, Giacomo Della Marca

**Affiliations:** 1 Department of Neurosciences, Università Cattolica del Sacro Cuore, Rome, Italy; 2 UOC Neurologia, Dipartimento Scienze dell’Invecchiamento, Neurologiche, Ortopediche e della Testa-Collo, IRCCS Fondazione Policlinico Universitario A. Gemelli, Rome, Italy; 3 UOC Neuroriabilitazione ad Alta Intensità, IRCCS Fondazione Policlinico Universitario A. Gemelli, Rome, Italy; 4 Department of Neurosciences, IRCCS Fondazione Policlinico Universitario A. Gemelli, Università Cattolica del Sacro Cuore, Largo A. Gemelli, 8, 00168 Rome, Italy

**Keywords:** Delirium, Stroke, Prognosis, Outcome, Disability, Mortality

## Abstract

**Introduction:**

Delirium is an acute fluctuating disorder of attention and awareness, which often complicates the clinical course of several conditions, including acute stroke. The aim of the present study was to determine whether delirium occurrence impacts the outcome of patients with acute stroke.

**Methods:**

The study design is single center, prospective, observational. We consecutively enrolled patients admitted to the stroke unit from April to October 2020. Inclusion criteria were age ≥ 18 years and diagnosis of acute stroke. Exclusion criteria were stroke mimics, coma, and terminal conditions. All patients were screened for delirium upon admission, within 72 h, and whenever symptoms suggesting delirium occurred by means of the Confusion Assessment Method for Intensive Care Unit and the Richmond Agitation Sedation Scale. Outcomes were evaluated with the 90-days modified Rankin Scale (mRS) by telephone interview.

**Results:**

The final study cohort consisted of 103 patients (62 men; median age 75 years, interquartile range 63–81). Thirty-one patients (30%) developed delirium. In the multivariate ordinal logistic regression, patients with delirium had higher mRS scores at 3 months (DLR + : mRS = 4 (3–6); DLR–: mRS = 1 (1–3); adjusted odds ratio = 4.83; CI = 1.88–12.35; *p* = 0.006). Delirium was a risk factor for death (mRS = 6) in the univariate logistic regression (OR 4.5, CI = 1.44–14.07; *p* = 0.010), but not in the adjusted analysis (OR 3.45; CI = 0.66–17.95; *p* = 0.142). Survival time during 90-days follow-up was shorter in the delirium group (Log Rank *χ*^2^ 3.89; *p* = 0.048).

**Conclusion:**

Delirium negatively impacts the prognosis of patients with acute stroke. Patients with post-stroke delirium have a worse functional outcome and a shorter survival.

## Introduction

Delirium is a neurobehavioral syndrome characterized by a disturbance of attention and awareness, which shows an acute onset and a fluctuating course. It represents a change from baseline mental status, and it is associated with disorders of cognition (such as memory deficit, disorientation, language, visuospatial ability, or perception) [1]. The diagnosis of delirium is clinical, and can be aided by several screening tools. Among these tools, the Confusion Assessment Method for Intensive Care Unit (CAM-ICU) is validated for use in critical care and in acute stroke patients [2, 3]. Delirium is a common neuropsychiatric complication of several healthcare settings, affecting as much as 50% of elderly hospitalized patients [4]. The occurrence rates of delirium in the acute phase of stroke vary across studies [5]. In a previous study on the same population, we observed an incidence of delirium in acute stroke patients of 30% [6].

Delirium has been extensively associated with adverse outcomes in terms of length of hospitalization [7], functional dependence [8, 9], and mortality [10, 11] in medical and surgery wards. However, limited studies have assessed the impact of delirium on the prognosis of stroke patients, providing inconsistent results [12–18].

The aim of the study was to determine the influence of delirium occurrence on the outcome of patients with acute stroke in terms of functional dependence and mortality, as expressed by modified Rankin Scale (mRS) scores at 90 days.

## Methods

### Patients

The study design was single center, prospective, observational. The study population was consecutively enrolled among patients admitted to the stroke unit of the Fondazione Policlinico Universitario Agostino Gemelli, Università Cattolica del Sacro Cuore, Rome, Italy. The present cohort is part of the population enrolled in a previous study on the incidence of delirium in stroke unit patients [6]. The enrollment period went on from April to October 2020. Inclusion criteria were age ≥ 18 years, diagnosis of ischemic or hemorrhagic stroke confirmed by CT or MRI scan, clinical onset of stroke in the previous 72 h, and a National Institute of Health Stroke Scale (NIHSS) score ≥ 1. The onset of symptoms was asked to the patient or to any witness. Exclusion criteria were transient ischemic attack (TIA), repeated negative CT or MRI scans, cerebral venous thrombosis, subarachnoid hemorrhage, coma, clinical conditions requiring intubation, and intensive care unit treatment.

Written informed consent was obtained from patients, or caregivers, at the time of enrollment. The study was performed in agreement with the Helsinki Declaration, and was approved by the Ethics committee of the Università Cattolica del Sacro Cuore.

### Demographic and clinical characteristics

All the patients underwent clinical assessment, including evaluation of demographic characteristics (sex and age), and determination of stroke-associated risk factors: atrial fibrillation (AF), hypertension, diabetes mellitus (DM), hypercholesterolaemia, heart diseases, and smoking habits. Comorbidities which could have impact on the occurrence of delirium, or on stroke prognosis, were assessed: thyroid disorders, chronic obstructive pulmonary disease (COPD), cancer, renal failure, cognitive impairment (evaluated on the basis of medical history or reported by relatives), and use of central nervous system (CNS) acting drugs. The burden of vascular leukoencephalopathy was assessed by means of the periventricular and deep Fazekas scores [19]. NIHSS score was determined repeatedly during admission in Stroke Unit; the NIHSS score referred to as ‘NIHSS at onset’ was the value measured at the time when the first CAM-ICU was administered.

Disability was measured with the mRS. The mRS ranges from 0 (no symptoms) to 6 (death). A score of 2 or less denotes functional independence [20]. Prestroke mRS score was established on admission.

### Diagnosis of delirium

Two validated diagnostic tools were used for the diagnosis of delirium: the Richmond Agitation Sedation Scale (RASS), and the CAM-ICU [2, 21, 22]. The scales were first administered at baseline, on admission, and 72 h later, or whenever patients developed symptoms suggesting delirium: fluctuation in mental status, altered consciousness, fluctuating attention, or disorganized thinking. Assessment of delirium was performed by the research team composed of neurology residents (ER, JM, and AC) and stroke physicians (GDM, GF, and AB), trained in delirium diagnosis.

**Fig. 1:**
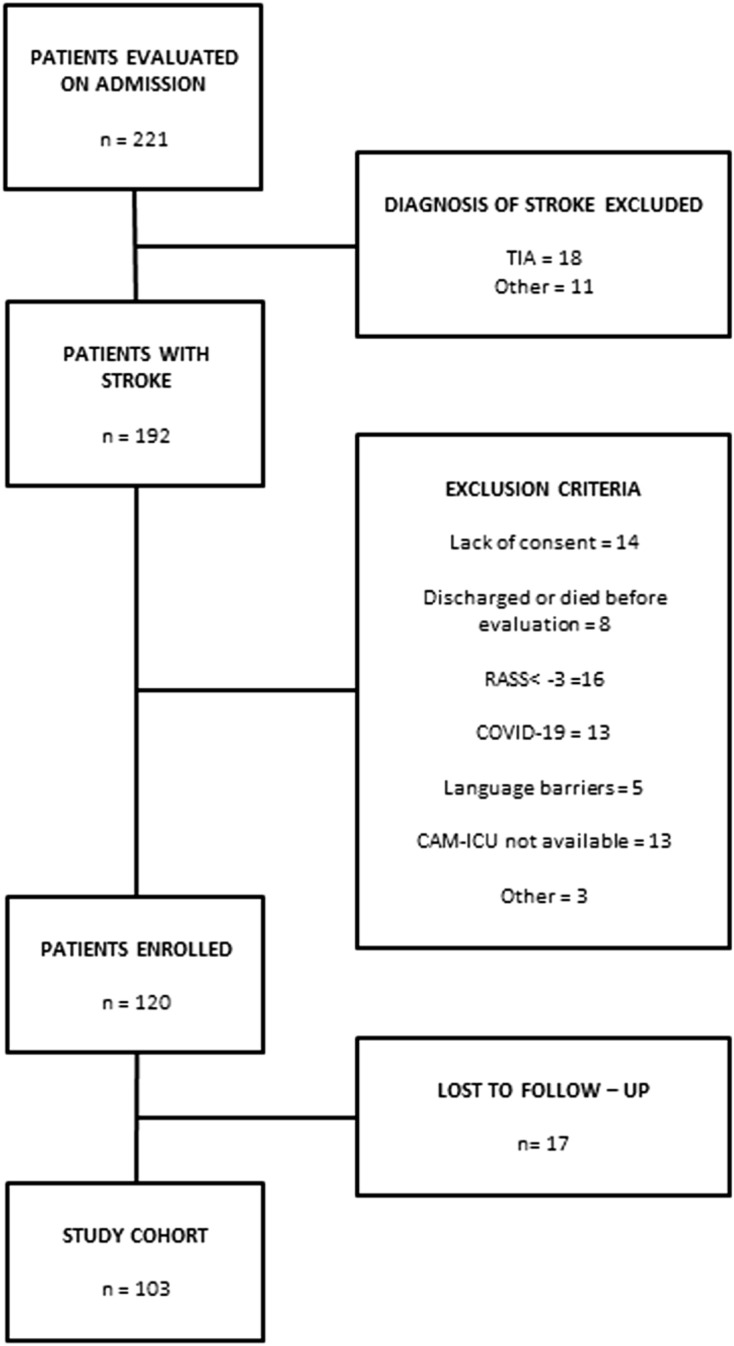
Study flow-chart. Abbreviations: *TIA* transient ischemic attack, *RASS* Richmond Agitation Sedation Scale, *CAM-ICU* Confusion Assessment Method for Intensive Care Unit

The RASS is validated [22] for the assessment and monitoring of sedation, especially in intensive care settings; the RASS score ranges from − 5 (unarousable) to 4 (combative) [22]. In the presence of delirium, it is helpful in classifying the motor subtypes of delirium: hyperactive, hypoactive, and mixed. Comatose patients, with RASS score < –3, were excluded from the study (Fig. 1). The CAM-ICU score is the most commonly used, validated [2, 3, 23] tool for the detection and monitoring of delirium. For a detailed description of the CAM-ICU scoring system in our cohort, see: Rollo et al., 2021 [6].

### Outcome assessment

Two study investigators (V.B. and I.S.), unaware of the group assignments, conducted a follow-up interview by telephone at 90 days with the patient, a proxy, or a health care provider. This interview provided reports for the assessment of the 90-days mRS.

The primary measure of outcome was the score on the mRS at 90 days.

Secondary outcomes were: the median time of hospitalization in stroke unit; the following dichotomizations of modified Rankin Scale: mRS 0–1 vs > 1, mRS 0–2 vs > 2, mRS 0–3 vs > 3; death (mRS = 6); survival time. A score of 2 or less was considered the cutoff for functional independence [20].

### Statistical analysis

The study sample size was calculated assuming outcome (mRS score) as primary endpoint variable. The G*Power [24, 25] software (version 3.1.9.6) was used for calculation. The study was planned for comparison between independent cases (patients with delirium versus patients without delirium), assuming a 30% of incidence of delirium in a series of patients admitted to stroke units [6] (allocation rate 0.3). The following settings were used: probability of a type I error < 0.05, effect size = 0.6, and power (1 − *β* error probability) = 0.7. The resulting sample size was 104 subjects.

Two groups of patients were compared: those who presented delirium (DLR +) and those who did not (DLR −). A list of the variables considered in the analysis is reported in Table 1. The primary effect variable was the adjusted common odds ratio for a shift in the direction of a worse outcome on the modified Rankin scale; this ratio was estimated with multivariate ordinal logistic regression. Dichotomic outcomes were analyzed with binary logistic regression and are reported as adjusted and unadjusted odds ratios with 95% confidence intervals. We calculated the odds ratios for possible cutoff values on the modified Rankin Scale (mRS = 0–1; mRS = 0–2; mRS = 0–3, and mRS = 6). To rule out the confounding effect of other prognostic variables, the common odds ratio and all secondary effect variables were adjusted for potential imbalances: age, pre-event mRS, NIHSS at onset, AF, DM, hypertension, and heart diseases. The Pearson and deviance tests were computed to test the goodness-of-fit of the ordinal logistic regression models. The *F*-test was performed to verify the goodness-of-fit of the linear regression model. The Hosmer–Lemeshow test and the Nagelkerke *R*^2^ were computed to test the goodness-of-fit of the multivariate binary logistic regression models. Threshold for significance was *p* < 0.05. All p values are two-sided. The adjusted and unadjusted common odds ratios, with 95% confidence intervals to indicate statistical precision, and the results of the goodness-of-fit analysis, are reported in Table 2.

**Table 1:** Demographic and clinical characteristics of the study cohort

Characteristics		Total study cohort (*n* = 103)	DLR + (*n* = 31)	DLR − (*n* = 72)
Male sex	*n* (%)	62 (60%)	18 (58%)	44 (61%)
Age—yr	Median (IQR)	75 (63–81)	77 (64–82)	75 (63–80)
Prestroke mRS	Median (IQR)	0 (0–1)	0 (0–2)	0 (0–0)
NIHSS	Median (IQR)	6 (3–13)	11 (6–18)	5 (2–10)
Large vessel occlusion	*n* (%)	43 (42%)	14 (45%)	29 (40%)
Thrombolysis	*n* (%)	27 (26%)	5 (16%)	22 (31%)
Thrombectomy	*n* (%)	31 (30%)	11 (35%)	20 (28%)
Hypertension	*n* (%)	77 (75%)	24 (77%)	53 (74%)
Atrial fibrillation	*n* (%)	29 (28%)	13 (42%)	16 (22%)
Diabetes mellitus	*n* (%)	24 (23%)	4 (13%)	20 (28%)
Renal failure	*n* (%)	5 (5%)	1 (3%)	4 (6%)
Heart diseases	*n* (%)	40 (39%)	9 (29%)	31 (43%)
Cognitive impairment	*n* (%)	4 (4%)	4 (13%)	0 (0%)
Deep Fazekas	Mean ± SD	1.17 ± 1.04	1.69 ± 1.04)	0.96 ± 0.98
Periventricular Fazekas	Mean ± SD	1.23 ± 1.02	1.48 ± 0.91	1.13 ± 1.06

Abbreviations: *yr* years, *IQR* interquartile range, *mRS* modified Rankin Scale, *NIHSS* National Institute of Health Stroke Scale, *DLR* + patients with delirium, *DLR–* patients without delirium

**Table 2:** Univariate and multivariate comparisons between patients with delirium and patients without delirium

	DLR + (*n* = 31)	DLR − (*n* = 72)	Effect variable	Unadjusted value (95% CI)	*p*	Adjusted value (95% CI)	*p*	Pearson (*p*)	Deviance (*p*)	*F*-test	Hosmer–Lemeshow (*p*)	Nagelkerke (*R*^2^)
Primary outcome												
mRS at 90-days median (IQR)	4 (3–6)	1 (1–3)	Odds ratio	6.80 (3.00–15.42)	*p* < 0.001	4.83 (1.88–12.35)	*p* = 0.006	*p* = 0.648	*p* = 1.00			
Secondary outcomes												
Length of hospitalization median (IQR)	12 (8–16)	7 (5–8)	Beta	7.15 (2.73–11.57)	*p* = 0.002	3.73 (1.27–6.19)	*p* = 0.003			*p* = 0.032		
mRS 0–1 at 90 days—*n* (%)	3 (10%)	38 (53%)	Odds ratio	10.43 (2.91–37.42)	*p* < 0.001	12.93 (2.50–66.96)	*p* = 0.002				*p* = 0.629	0.436
mRS 0–2 at 90 days—*n* (%)	7 (23%)	49 (68%)	Odds ratio	7.30 (2.75–19.40)	*p* < 0.001	5.93 (1.73–20.33)	*p* = 0.005				*p* = 0.707	0.438
mRS 0–3 at 90 days—*n* (%)	12 (39%)	57 (79%)	Odds ratio	6.02 (2.40–15.09)	*p* < 0.001	4.78 (1.39–16.45)	*p* = 0.013				*p* = 0.860	0.406
Death—mRS 6 at 90 days—*n* (%)	9 (29%)	6 (8%)	Odds ratio	4.5 (1.44–14.07)	*p* = 0.010	3.45 (0.66–17.95)	*p* = 0.142				*p* = 0.001	0.409

The covariates considered in the adjusted analysis were: age, pre-event mRS, NIHSS at onset, atrial fibrillation, diabetes mellitus, hypertension, and heart diseases

Abbreviations: *mRS* modified Rankin Scale, *IQR* interquartile range, *DLR* + patients with delirium, *DLR–* patients without delirium

Kaplan–Meier curves were used to present mortality according to post-stroke delirium incidence. Log Rank test was used to compare survival time between the DLR + and DLR − groups.

Numerical variables are presented as median and interquartile range (IQR); categorical variables are presented as number (*n*) and percentage.

All statistics were performed by means of the Statistical Package for Social Science (SPSS^®^) software, version 22 (SPSS^®^, Inc., Chicago, IL, USA).

## Results

A CONSORT diagram depicting the enrollment process is reported in Fig. 1. Starting from 221 patients admitted to the stroke unit in the study period, after screening for inclusion and exclusion criteria, 120 patients were included. In this population, the overall incidence of delirium was 36/120 (30%) [6]. Of the 120 patients included, 17 patients were lost at the 3-months follow-up (Fig. 1). Patients lost to follow-up did not differ significantly from the final cohort concerning all the baseline study variables, including delirium incidence. The final study cohort consisted of 103 patients (62 men; age median 75 years, IQR 63–81). Among the patients in the study cohort, 31/103 (30%) patients developed delirium in the acute phase of stroke. Demographic and clinical features of the study cohort, and of the subgroups with and without delirium, are listed in Table 1.

**Fig. 2:**
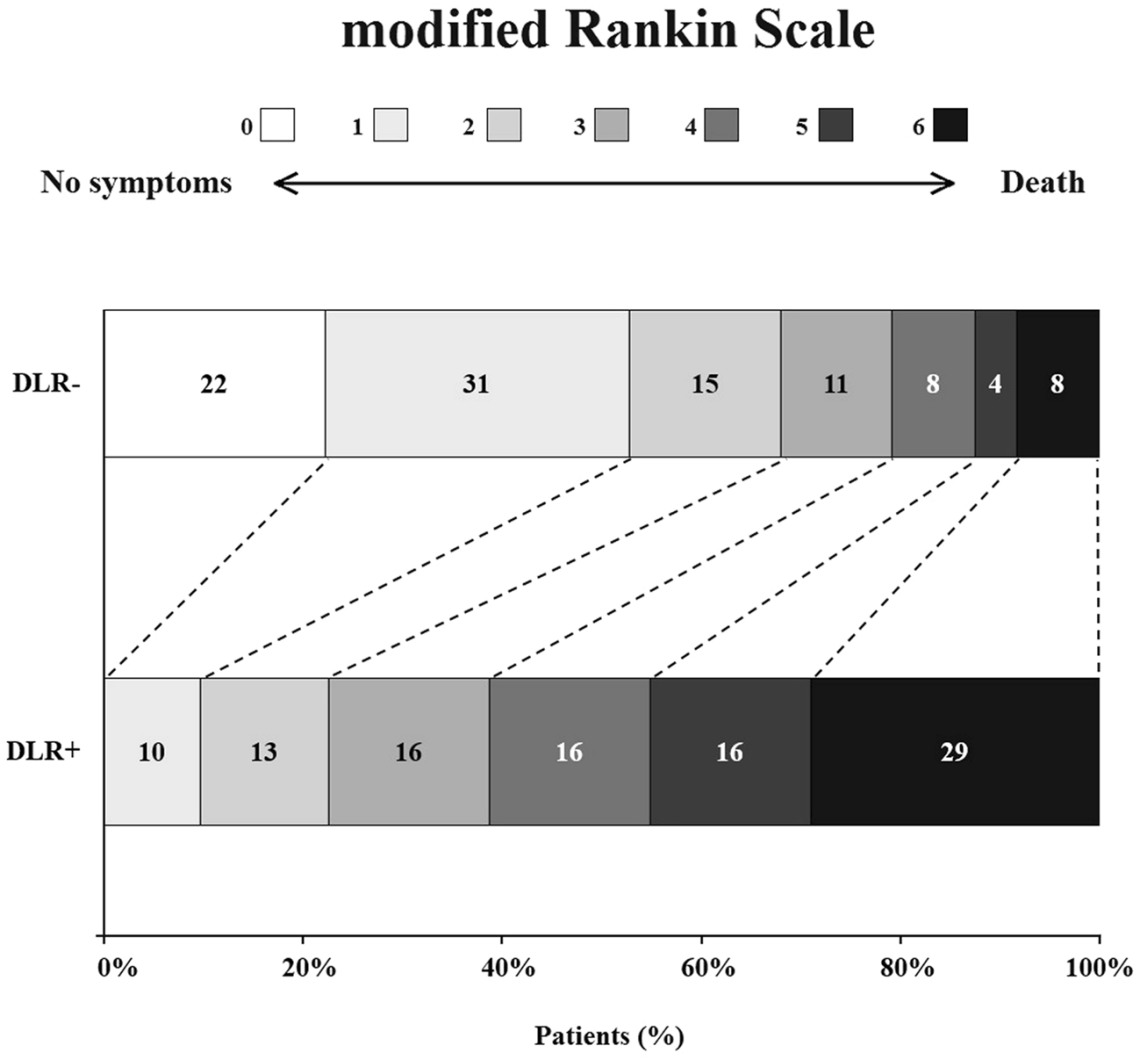
Modified Rankin Scale scores distribution in the two subgroups. Numbers in the histograms indicate the percentage of patients for each mRS score. Abbreviations: *DLR −* patients without delirium, *DLR +* patients with delirium. Scores range from 0 to 6, with 0 indicating no symptoms, 1 some symptoms, but no significant disability, 2 slight disability (patient is able to look after own affairs without assistance but is unable to carry out all previous activities), 3 moderate disability (patient requires some help but is able to walk unassisted), 4 moderately severe disability (patient is unable to attend to own bodily needs without assistance and unable to walk unassisted), 5 severe disability (patient bedridden, requires constant nursing care and attention), and 6 death

In the univariate ordinal logistic regression, patients with delirium had higher mRS scores at 3 months (DLR + : mRS = 4 (3–6); DLR −: mRS = 1 (1–3); OR = 6.80; CI = 3.00–15.42; *p* < 0.001). In the delirium group, there was a shift in the distribution of the mRS scores towards a worse outcome (Fig. 2). In the multivariate ordinal logistic regression, delirium was an independent predictor of poor outcome (adjusted odds ratio = 4.83; CI = 1.88–12.35; *p* = 0.006), after adjustment for possible confounders (age, NIHSS, pre-stroke mRS, hypertension, AF, DM, and heart diseases).

**Fig. 3:**
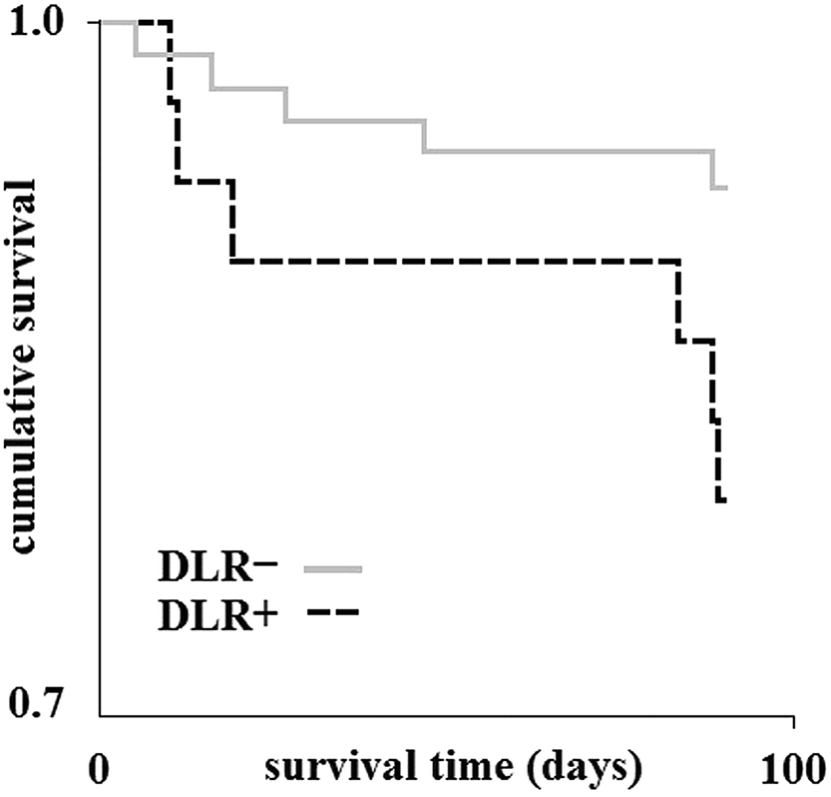
Kaplan–Meier curves demonstrating survival time in patients with and without post-stroke delirium in the 90-days interval. *DLR +* patients with delirium; *DLR −* patients without delirium

As concerns the secondary outcomes, median time of hospitalization was higher in patients with delirium in unadjusted comparison (DLR + 12 days (8–16); DLR −: 7 days (5–8); Beta: 7.15; CI = 2.73–11.57; *p* = 0.002) and after adjustment (Beta 3.73; CI = 1.27–6.19; p = 0.003). Patients without delirium had higher odds of being in all the subgroups with better functional outcome: mRS 0–1 (OR 10.43; CI = 2.91–37.42; *p* < 0.001); mRS 0–2 (OR 7.30; CI = 2.75–19.40; *p* < 0.001); mRS 0–3 (OR 6.02; CI = 2.40–15.09; *p* < 0.001). These differences were still statistically significant after adjustment for the pre-specified variables. Delirium was found to be a risk factor for death (mRS = 6) in the univariate logistic regression (OR 4.5, CI = 1.44–14.07; *p* = 0.010). The adjusted odds ratio did not reach significant difference (OR 3.45; CI = 0.66–17.95; *p* = 0.142). Nevertheless, mean survival time in the 90-days follow-up was shorter in patients with delirium (DLR + : 84 ± 20 days; DLR −: 87 ± 13 days; Log Rank *χ*^2^: 3.89; *p* = 0.048): at 90 days 27/31 (87.1%) patients with delirium were alive, versus 69/72 (95.8%) patients without delirium. Results of the unadjusted and adjusted comparisons are reported in Table 2. Cumulative survival and survival time are represented with Kaplan–Meier curves (Fig. 3).

## Discussion

The primary outcome of our study was to assess the impact of delirium occurrence in the acute phase of stroke on the prognosis at 90 days. Delirium is independently associated with a worse outcome, as expressed by a shift towards higher modified Rankin Scale scores. Moreover, delirium carries out increased risk of disability and functional dependence after three months from acute stroke. These findings were confirmed after adjustment for several relevant confounders, such as age, severity of stroke, pre-event disability, and comorbidities.

Overall, our results are in line with the existing literature, even if previous studies are highly heterogeneous concerning the scales used for the diagnosis of delirium, the assessment of functional outcome and the timeframe point considered. Similar results are reported by a recent study, which showed that delirium occurred in 31% of patients with acute stroke, and it was an independent predictor of mRS > 2 at 90 days [18]. In a prospective observational study, Oldenbeuving et al. evaluated functional outcome after 1 month from the acute phase of stroke with the Barthel Index (BI), showing that BI was worse in stroke survivors with delirium [12]. Qu et al. found a worse functional outcome, as expressed by the Lawton Instrumental Activities of Daily Living (IADL), in patients with post-stroke delirium evaluated at 3-months follow-up [15]. In a study by Miu et al., delirium was associated with a worse prognosis as reflected by higher mRS and lower BI at discharge, 6-months and 12-months follow-ups [14].

As concerns the other secondary outcomes, patients with post-stroke delirium had a 3.7-fold increased risk of prolonged hospital stay: hospitalization in patients with delirium was 5 days longer than in patients without delirium. Such finding confirms results from previous studies [12, 14]. In our study, patients with delirium showed increased risk of death compared to controls, even if such difference did not reach statistical significance after adjustment for potential confounders.

Previous studies assessing prognosis of stroke survivors over a longer period (up to 6–12 months) found conflicting evidence regarding impact of delirium on mortality [13, 14, 16]. Delirium was significantly associated with mortality rate at 6 and 12 months after stroke, but not with mortality at 1 month in a study by Sheng et al. [16]. On the other hand, McManus et al. found significantly higher in-patient mortality in the delirium group, whereas mortality at 1-year follow-up was not different between the delirium and the control group [13]. In a recent study on a large cohort of Intensive Care Unit patients, who underwent a 2.5-years follow-up, delirium was associated only with mortality at 30-days post-hospital discharge [26]. It must be taken into account that our assessment of mortality was performed at 90 days after the index event (i.e., acute stroke onset).

Survival time in our cohort was shorter in patients displaying delirium in the acute phase of stroke (Fig. 3). This result is coherent with findings from a previous study conducted over a large cohort of stroke patients, which showed reduced survival time in patients with delirium at 3 and 12 months following acute stroke [17].

To date, the best quality of evidence on the topic comes from a systematic review and metanalysis, including 10 studies with a total of 2004 patients with acute stroke, which reported that patients with delirium had higher probability to be discharged to institutions, higher length of hospitalization, and higher in-hospital mortality [27]. However, the main limitation arising from this meta-analysis concerns the lack of adjustment for relevant confounders, such as severity of stroke and comorbidities, known to influence stroke outcomes. More recent studies [17, 18], which addressed the adjustment for relevant confounders in their multivariate models, found a significant association of stroke severity [17, 18], age [17], and absence of thrombolytic treatment [18] with worse outcome. Moreover, in the study by Pasińska et al. [17], patients with delirium had more severe concomitant chronic diseases (in particular atrial fibrillation and diseases affecting respiratory tract), even if these factors were not included in the multivariate model. In our study, delirium was the only predictor of worse prognosis after acute stroke, since all the other confounders which were included in our multivariate model (namely, age, NIHSS on admission, atrial fibrillation, hypertension, heart diseases, and diabetes) were not significantly associated with poor outcome. Thus, such finding adds knowledge to the impact of delirium as a strong predictor of stroke outcome.

Since the publication of the meta-analysis [27], the clinical scenario of ischemic stroke radically changed, due to the validation of mechanical thrombectomy as an effective revascularization treatment [28]. This is particularly meaningful when addressing the topic of stroke outcome, since the prognosis of a stroke due to large vessel occlusion is also driven by the efficacy of the revascularization treatment. Our study was conducted in this new clinical scenario, and included nearly one-third of patients treated with mechanical thrombectomy (Table 1).

The present study has some limitations. The main limitation of our study is the relatively small cohort of patients enrolled. Moreover, we aimed to evaluate delirium in acute stroke patients during the time of stay in stroke unit; therefore, some cases of late-onset delirium may have been missed. Finally, we did not evaluate outcome by means of a thorough neurological examination at 3 months. Even so, the modified Rankin Scale is a reliable tool, with a satisfactory inter-observer agreement, extensively used in clinical trials to evaluate the outcome of stroke patients [20].

A strength of our study was the highly homogenous study cohort: we did not include conditions which could bias the evaluation of delirium incidence and patient’s prognosis. Indeed, we did not include patients with TIA, coma, extremely severe conditions, and dementia. We prospectively evaluated patients with repeated daily delirium assessments, by means of a largely employed and validated tool. Moreover, there was no significant difference concerning age and pre-stroke disability between our delirium and control groups. This reinforces the role of delirium impact on the outcome of patients, as confirmed by the adjusted statistical comparison.

In conclusion, our study found that delirium negatively impacts the prognosis of patients with stroke. Patients with post-stroke delirium had a worse functional outcome and a shorter survival. Data suggest that delirium has an impact on the outcome of stroke, but this issue needs to be confirmed by larger studies and possibly by a state-of the-art systematic review and meta-analysis. Indeed, in the last few years, the therapeutic scenario of acute stroke has changed due to the introduction of mechanical thrombectomy and the extension of the therapeutic window for both intravenous and endovascular treatment. Therefore, further studies are needed to clarify the impact of delirium on acute stroke outcome, given the modification of stroke prognosis due to better treatment chances. Early recognition of delirium and employment of treatment strategies, which do not exclusively encounter pharmacological therapy, are paramount needs in acute stroke management. Besides this, the adoption of an effective delirium preventive protocol in all stroke units is crucial to improve stroke patient’s prognosis.

## References

[1] Association AP (2013) Diagnostic and statistical manual of mental disorders (DSM-5®). American Psychiatric Pub. https://www.psychiatry.org/psychiatrists/practice/dsm 10.1590/s2317-17822013000200017 24413388

[2] Ely EW, Margolin R, Francis J (2001). Evaluation of delirium in critically ill patients: validation of the Confusion Assessment Method for the Intensive Care Unit (CAM-ICU). Crit Care Med.

[3] Fleischmann R, Warwas S, Andrasch T (2021). Course and recognition of poststroke delirium: a prospective noninferiority trial of delirium screening tools. Stroke.

[4] Inouye SK, Westendorp RGJ, Saczynski JS (2014). Delirium in elderly people. Lancet Lond Engl.

[5] Shaw RC, Walker G, Elliott E, Quinn TJ (2019). Occurrence rate of delirium in acute stroke settings: systematic review and meta-analysis. Stroke.

[6] Rollo E, Callea A, Brunetti V (2021). Delirium in acute stroke: a prospective, cross-sectional, cohort study. Eur J Neurol.

[7] Ely EW, Shintani A, Truman B (2004). Delirium as a predictor of mortality in mechanically ventilated patients in the intensive care unit. JAMA.

[8] Rudolph JL, Inouye SK, Jones RN (2010). Delirium: an independent predictor of functional decline after cardiac surgery. J Am Geriatr Soc.

[9] Siddiqi N, House AO, Holmes JD (2006). Occurrence and outcome of delirium in medical in-patients: a systematic literature review. Age Ageing.

[10] Witlox J, Eurelings LSM, de Jonghe JFM (2010). Delirium in elderly patients and the risk of postdischarge mortality, institutionalization, and dementia: a meta-analysis. JAMA.

[11] Seiler A, Blum D, Deuel JW (2021). Delirium is associated with an increased morbidity and in-hospital mortality in cancer patients: results from a prospective cohort study. Palliat Support Care.

[12] Oldenbeuving AW, de Kort PLM, Jansen BPW (2011). Delirium in the acute phase after stroke: incidence, risk factors, and outcome. Neurology.

[13] Mc Manus JT, Pathansali R, Ouldred E (2011). Association of delirium post-stroke with early and late mortality. Age Ageing.

[14] Miu DKY, Yeung JCY (2013). Incidence of post-stroke delirium and 1-year outcome. Geriatr Gerontol Int.

[15] Qu J, Chen Y, Luo G (2018). Delirium in the acute phase of ischemic stroke: incidence, risk factors, and effects on functional outcome. J Stroke Cerebrovasc Dis.

[16] Az S, Shen Q, Cordato D (2006). Delirium within three days of stroke in a cohort of elderly patients. J Am Geriatr Soc.

[17] Pasińska P, Wilk A, Kowalska K (2019). The long-term prognosis of patients with delirium in the acute phase of stroke: PRospective Observational POLIsh Study (PROPOLIS). J Neurol.

[18] Taiuan FSI, Pedro AL, Tiago TA (2021). Impact of delirium and its motor subtypes on stroke outcomes. Stroke.

[19] Fazekas F, Barkhof F, Wahlund LO (2002). CT and MRI rating of white matter lesions. Cerebrovasc Dis Basel Switz.

[20] van Swieten JC, Koudstaal PJ, Visser MC (1988). Interobserver agreement for the assessment of handicap in stroke patients. Stroke.

[21] De J, Wand APF (2015). Delirium screening: a systematic review of delirium screening tools in hospitalized patients. Gerontologist.

[22] Sessler CN, Gosnell MS, Grap MJ (2002). The Richmond Agitation-Sedation Scale: validity and reliability in adult intensive care unit patients. Am J Respir Crit Care Med.

[23] Mitasova A, Kostalova M, Bednarik J (2012). Poststroke delirium incidence and outcomes: validation of the Confusion Assessment Method for the Intensive Care Unit (CAM-ICU). Crit Care Med.

[24] Faul F, Erdfelder E, Buchner A, Lang A-G (2009). Statistical power analyses using G*Power 3.1: tests for correlation and regression analyses. Behav Res Methods.

[25] Faul F, Erdfelder E, Lang A-G, Buchner A (2007). G*Power 3: a flexible statistical power analysis program for the social, behavioral, and biomedical sciences. Behav Res Methods.

[26] Fiest KM, Soo A, Hee Lee C (2021). Long-term outcomes in intensive care unit patients with delirium: a population-based cohort study. Am J Respir Crit Care Med.

[27] Shi Q, Presutti R, Selchen D, Saposnik G (2012). Delirium in acute stroke: a systematic review and meta-analysis. Stroke.

[28] Berkhemer OA, Fransen PSS, Beumer D (2015). A randomized trial of intraarterial treatment for acute ischemic stroke. N Engl J Med.

